# Measuring diagnostic safety of inpatients: time to set sail in uncharted waters

**DOI:** 10.1515/dx-2015-0003

**Published:** 2015-02-04

**Authors:** Viraj Bhise, Hardeep Singh

**Affiliations:** Houston Veterans Affairs Center for Innovations in Quality, Effectiveness and Safety, Michael E. DeBakey Veterans Affairs Medical Center and the Section of Health Services Research, Department of Medicine, Baylor College of Medicine, Houston, TX, USA; and Division of Management, Policy and Community Health, School of Public Health, University of Texas Health Science Center at Houston, TX, USA; Houston Veterans Affairs Center for Innovations in Quality, Effectiveness and Safety, Michael E. DeBakey Veterans Affairs Medical Center and the Section of Health Services Research, Department of Medicine, Baylor College of Medicine, Houston, TX, USA

The patient safety world was recently given reasons to be optimistic. A report by the U.S. Department of Health and Human Services (HHS) measured the rate of Hospital-Acquired Conditions (HACs) in patients admitted to US hospitals, and estimated that 50,000 fewer patients died as a result of a reduction in HACs from 2010 to 2013 [1]. This impressive quantitative improvement has been largely attributed to the Partnership for Patients (PfP) initiative led by the Center for Medicare and Medicaid Services (CMS). The PfP initiative focuses on making hospitals safer, more reliable and less costly and has identified ten core patient safety areas to improve inpatient quality of care and reduce readmissions [2]. It has developed measures to monitor hospital acquired infections including surgical site, blood-stream and ventilator associated infections, adverse drug events, in-hospital patient falls, pressure ulcers and venous thromboembolism in participating healthcare organizations. While this decrease in adverse events over the last 3 years is encouraging, we must be cautious in our interpretation [3]. The PfP initiative and other ongoing national efforts do not include or address diagnostic errors, even though they are increasingly concerning for patients and estimated to contribute to substantive harm [4]. Challenges in identifying, defining and measuring diagnostic errors in the hospital setting are partly responsible for why national efforts are unable to focus on them.

In this issue of *Diagnosis*, Shenvi and El-Kareh [5] review the literature on criteria to find diagnostic errors in hospitalized patients. They find several criteria potentially related to inpatient diagnostic errors that might serve as a starting point for automated detection of errors using trigger tools. Because triggers use specific clues to select a high-risk cohort of patients for record reviews, they can be useful to jumpstart the measurement and study of inpatient diagnostic error. Triggers are a bit analogous to picking out needles (errors) from a haystack (vast numbers of patient records), by using techniques to make the haystack smaller. If inpatient triggers for diagnostic errors can be developed, it will add a new dimension to study diagnostic errors, because thus far only outpatient diagnostic errors have been subjected to any trigger-related work [6, 7].

The authors categorize triggers into a framework based on four clinical situations potentially related to diagnostic error – patient deterioration, unexpected time course of illness, change of management plan and diagnostic uncertainty. While most triggers listed in the article were used previously for retrospective detection of error, some of them have the potential to be used prospectively or in a near real-time manner. This approach is similar to the outpatient setting where selective record reviews have been conducted either based on return visits to identify errors retrospectively or based on missed follow-up of test results to identify errors prospectively [6, 7]. With new inpatient triggers, we could build a comprehensive strategy to cover the diagnostic process through multiple lenses [8].

Despite this optimistic note, much remains to be done to bring these triggers to real-world inpatient practice. The reliability and validity of these triggers to detect inpatient diagnostic error will partly determine their success but are currently unknown. We do not have knowledge of individual positive predictive values (PPVs) for these triggers and thus rigorous validation by researchers is needed to advance the science in this area. There is little funding infrastructure currently to stimulate research to develop and test such triggers to make them sensitive and specific. Additionally, measurement in an electronic health record (EHRs)-based healthcare environment is challenging as most institutions have not yet set up robust repositories of clinical EHR data that can be queried and analyzed. And even when we are able to develop reliable and valid measurement tools, hospitals might not use them. Currently, hospitals are under several different types of competing pressures and priorities including meeting quality and safety measure requirements that are unrelated to diagnosis [9]. Hospitals not only need good tools and strategies to measure diagnostic safety but also need incentives to integrate diagnostic error into their existing patient safety programs [10]. It will take major policy shifts and culture change for hospitals to monitor and measure inpatient diagnostic error.

The year 2015 is one full of high expectations as we look forward to the Institute of Medicine report on diagnostic error [11]. Nevertheless, measurement of diagnostic error will continue to pose several fundamental challenges for healthcare organizations and would be a major threat to advancements in patient safety. The review by Shenvi and El-Kareh is a useful stepping stone for future research to validate clinical criteria for triggers to screen for inpatient diagnostic error. More research is called for to develop and test triggers with high PPVs that can be implemented in hospitals for routine monitoring, measurement and improvement. It will be a bit premature to celebrate successes in patient safety without accounting for diagnostic errors so it is time we start making progress in these uncharted waters.
